# Prednisolone Co-Administered with Losartan Confers Renoprotection in Patients with IgA Nephropathy

**DOI:** 10.1080/08860220701260511

**Published:** 2007-05-10

**Authors:** Yoshio Horita, Masato Tadokoro, Kouichi Taura, Ryuichi Ashida, Mayumi Hiu, Takashi Taguchi, Akira Furusu, Shigeru Kohno

**Affiliations:** Division of Nephrology, Department of Medicine, National Hospital Organization, Nagasaki Medical Center; and Department of Internal Medicine, Nagasaki Municipal Medical Center, Nagasaki, Japan; Department of Internal Medicine, Nagasaki Municipal Medical Center, Nagasaki, Japan; Division of Nephrology, Department of Medicine, National Hospital Organization, Nagasaki Medical Center, Nagasaki, Japan; Department of Pathology, Nagasaki University Graduate School of Biomedical Sciences, Nagasaki, Japan; Department of Internal Medicine, Nagasaki University Graduate School of Biomedical Sciences, Nagasaki, Japan

**Keywords:** corticosteroid, angiotensin II receptor blockers, Immunoglobulin A, nephropathy

## Abstract

**Background:**

Treatment options for progressive IgA nephropathy are limited.

**Methods:**

We performed a small, randomized controlled trial to evaluate the effects of prednisolone (PSL, 30 mg/dL, gradually tapered to 5 mg/dL over two years) plus 50 mg/day of losartan (LST, an angiotensin II receptor blocker) or PSL alone on IgA nephropathy. We separated 38 patients (age, 33 ± 11 years; creatinine clearance, 103 ± 31 mL/min; proteinuria, 1.6 ± 0.5 g/day) into two groups that were treated with either PSL plus LST or PSL alone, and compared the proteinuria and creatinine clearance after two years. Baseline and histopathological data did not significantly differ between the two groups.

**Results:**

Two years of treatment in both groups significantly decreased proteinuria compared with baseline, and PSL plus LST (from 1.6 ± 0.6 to 0.3 ± 0.1 g/day, *p* < 0.05) was more effective than PSL alone (from 1.6 ± 0.3 to 0.5 ± 0.1 g/day, *p* < 0.05). Creatinine clearance in both groups was similar at the start of study but significantly differed at the end of the study (PSL plus LST, 104.3 ± 36.4 to 100.4 ± 38.9 mL/min; PSL alone, 103.4 ± 28.5 to 84.8 ± 34.3 mL/min, *p* < 0.05).

**Conclusions:**

Combined therapy with PSL plus LST appears to be more effective than PSL alone in reducing proteinuria and protecting renal function in patients with IgA nephropathy.

## INTRODUCTION

Immunoglobulin A nephropathy (IgAN) is the most prevalent type of primary glomerulonephritis worldwide and in Japan.[1^,^2] Among the clinical and laboratory features, hypertension and proteinuria are important predictors of a poor outcome.[3] Proteinuria causes renal damage that leads to the more rapid progression of renal diseases toward end-stage renal failure.[4] Because angiotensin II plays a critical role in the pathogenesis of proteinuria, angiotensin-converting enzyme (ACE) inhibitors are often administered to patients with proteinuric nephropathies.[4^–^6] Among the many factors that affect the progression of IgAN, blood pressure (BP) is one of the most important.[7] However, although antihypertensive approaches applied to humans and other experimental animals with chronic kidney diseases (CKD) have proven positive, the effects of such treatment on the initial stage of IgAN remain unknown. Some major trials of antihypertensive therapy in renal diseases with proteinuria have suggested that optimal BP control protects renal function.[8] A recent study has shown that ACE inhibitors and angiotensin II receptor blockers (ARBs) can reduce glomerular hypertension, proteinuria, and glomerular size-selectivity,[9] and clinical trials have shown that these drugs also slow the progression of CKD.[4^–^7] Some randomized clinical trials and retrospective cohort studies of patients with IgAN have found that ACE inhibitors and/or ARBs moderately lower urinary protein excretion.[9^–^11] However, whether or not ACE inhibitors and ARBs preserve renal function in patients with IgAN remains obscure.[8^–^10] Moreover, the early initiation of antihypertensive treatment for IgAN has still not been investigated.

Corticosteroids have been used for more than 20 years to treat IgAN because of their anti-inflammatory and immunosuppressive properties. An ideal protocol of steroid therapy is required that can prevent the progression of IgAN with minimal toxicity. Katafuchi et al.[11] have recently described a randomized, controlled trial of 90 adult IgAN patients who received either supportive therapy or low dose prednisolone for two years. Prednisolone lowered proteinuria after two years but did not improve renal survival. Therefore, they suggested that the low dose of PSL in the protocol might have caused the discrepancy between the effects on proteinuria and renal survival.[11] Moreover, two other randomized trials of small patient cohorts revealed that steroids similarly decreased proteinuria but did not affect renal function.[12^,^13] Because we found that both PSL and ARBs reduced proteinuria in IgAN, we questioned whether a combination of PSL and ARBs in the setting of normotensive and moderately proteinuric IgAN with moderate histological features could improve the anti-proteinuric effect and preserve renal function better than PSL alone.

## SUBJECTS AND METHODS

This prospective, randomized controlled clinical trial proceeded in accordance with the standards of the Ethics Committee of Nagasaki Municipal Medical Center, and all patients provided written, informed consent to participate.

### Patients

Between 2000 and 2003, IgAN was newly diagnosed in 66 of 189 biopsies at Nagasaki Municipal Medical Center. Of 66 patients with IgAN, 40 patients satisfied the following criteria and were enrolled in the trial. Entry criteria were normal BP of <140/90 mmHg; mean arterial pressure <107 mmHg, persistent mild to moderate proteinuria of 1.6 ± 0.5 g/day (range, 1.0–2.6), and normal or mild to moderately reduced but stable renal function (creatinine clearance; Ccr >50 mL/min/1.73m^2^), with a renal glomerular score between 4 and 7 according to Katafuchi's scale.[14] Patients with systemic diseases such as diabetes, lupus erythematosus, chronic liver diseases, renal allografts, and Henoch-Schönlein purpura were excluded. The patients did not receive ACE inhibitors, ARBs, immunosuppressive therapy, or diuretic and statin treatment throughout the study.

### Administration of PSL Plus LST

The angiotensin II receptor blocker losartan (LST) was combined with PSL and administered to 22 patients with IgAN, and PSL alone was given to another 18. Two dropped out of the study due to postural hypotension. All statistical calculations were thus obtained from data generated by 38 patients (18 females and 20 males; age, 33.5 ± 11.0 years; range, 16–52 years), who were randomly assigned to the PSL or combined group. Twenty patients aged 34.6 ± 12.0 (8 females and 12 males) received PSL orally at sequential doses of 30 mg/dL for two months, 25 mg/dL for two months, 20 mg/dL for two months, 15 mg/dL for six months, 10 mg/dL for twelve months, and 5 mg/dL for one month, together with 50 mg of LST daily. Eighteen patients aged 32.3 ± 10.6 (10 females and 8 males) received PSL alone over a period of 24 months. Dipyridamole (300 mg/dL) was administered to both groups.

### Clinical and Laboratory Procedures

Complete medical histories were obtained and physical examinations were performed on all patients to obtain baseline data at the start of the study. Side effects were examined, and the patients were questioned about their symptoms once each month during follow-up. The patients were instructed to take the medication once after awakening every morning, and blood samples were tested 3 h later. Baseline laboratory tests included a complete blood count, serum chemistry, and 24-h urine collection for protein. Blood pressure and serum creatinine were tested again one week after starting the protocol. The patients remained on their usual diet (free intake of salt and protein) for the duration of the study. The treatment effects were prospectively assessed monthly. The patients were seated for BP and heart rate measurements. Protein excretion was evaluated in 24-h urine samples after 6, 12 and 24 months. Serum and urinary values were measured after 6, 12, and 24 months by routine laboratory methods using a JEOL JCA-BM1650 autoanalyzer (JEOL, Tokyo, Japan). We evaluated the Ccr after 6, 12, and 24 months using the Gault-Cockcroft equation.[15] The same clinician measured BP in seated patients once each after at least 15 min of rest, using a standard sphygmomanometer with an appropriately sized cuff. The values obtained from three measurements at 3 min intervals were averaged. The first and fifth Korotkoff sounds were taken as systolic and diastolic BP (SBP and DBP), respectively. Mean arterial pressure (MAP) was calculated as the sum of one-third of the SBP and two-thirds of the DBP.

### Pathological Examination

Glomerular, interstitial, and vascular lesions were scored by assessing samples stained with hematoxylin and eosin, periodic acid-Schiff, trichrome, and silver, using light microscopy, immunofluorescence, and electric microscopy. The immunofluorescence findings were classified between none (−) and 3+ according to the intensity of fluorescence of each specimen. The diagnosis was limited to primary mesangial proliferative glomerulonephritis with a predominant deposition of IgA in the mesangium. The specific pathological features of the patients were scored by light microscopy as described by Katafuchi et al.[14] Glomerular lesions (total score, 0–12) included glomerular hypercellularity, glomerular segmental types, and glomerular sclerosis. The score for each glomerular lesion was as fellows: 0, none; 1, <10% of glomeruli involved; 2, 10– 24%; 3, 25–49%; 4, 50+% of glomeruli involved. The severity of interstitial cell infiltration, interstitial fibrosis, tubular atrophy, arterial wall thickening, and arteriolar hyaline change in each patient was semi-quantified (total score, 0–9) from 0 to 3 according to the ratio (%) of injured tissue: 0, none; 1, <25%; 2, 25–49%; 3, 50+%.

### Statistical Analysis

All data are expressed as means ± SD. We applied an unpaired Student's t-test to determine the significance of differences among the three treatment groups. Discrete data were examined using the X^2^ analysis. Values of *p* < 0.05 were considered to indicate statistical significance.

## RESULTS

### Baseline Data

Twenty patients received PSL plus LST and 18 patients received PSL alone for 24 months. The two groups did not significantly differ in terms of age, gender, BP, baseline laboratory values (see Table 1) or Katafuchi's grading of renal lesions at baseline (see Table 2).

**Table 1:** Comparison of baseline characteristics between patient groups

Characteristic	PSL + LST (n=20)	PSL (n = 18)	*p*
Male/female	12/8	8/10	0.53
Age, y	34 ± 12	32 ± 10	0.67
BMI, kg/m^2^	21.2 ± 3.1	19.9 ± 1.7	0.30
Systolic BP, mmHg	120 ± 8	121 ± 7	0.90
Diastolic BP, mmHg	73 ± 6	75 ± 7	0.59
Heart rate, beats/min	71 ± 4	72 ± 6	0.78
Na, mEq/L	142.5 ± 1.4	141.2 ± 1.4	0.65
K, mEq/L	4.3 ± 0.3	4.4 ± 0.2	0.68
BUN, mg/dL	14.1 ± 4.4	14.7 ± 3.7	0.75
sCr, mg/dL	0.8 ± 0.2	0.7 ± 0.1	0.52
Ccr ml/min/1.73 m^2^	104 ± 36	103 ± 28	0.95
Proteinuria, g/24 h	1.6 ± 0.6	1.6 ± 0.4	0.75
Serum IgA, mg/dL	330 ± 75	366 ± 131	0.47

Abbreviations: PSL = prednisolone, LST = losartan, BMI = body mass index, BP = blood pressure, BUN = blood urea nitrogen, sCr = serum creatinine, Ccr = creatinine clearance, IgA = immunoglobulin A.

**Table 2:** Comparison of baseline histopathologic features between patient groups

Characteristic	PSL + LST (n = 20)	PSL (n = 18)	*p*
Glomerular hypercellularity	1.3 ± 0.4	1.4 ± 0.5	0.29
Glomerular segmental lesions	1.5 ± 0.5	1.5 ± 0.5	0.50
Glomerular global sclerosis	1.0 ± 0.9	1.1 ± 0.8	0.43
Cellular (fibrocellular) crescents	0.5 ± 0.7	0.7 ± 0.9	0.49
Tuft adhesion	0.9 ± 0.3	1.1 ± 0.6	0.36
Glomerular score	5.2 ± 0.5	6.0 ± 0.6	0.34
Interstitial cell infiltration	1.3 ± 0.6	1.5 ± 0.7	0.44
Interstitial fibrosis	1.1 ± 0.7	1.2 ± 0.8	0.74
Tubular atrophy	1.1 ± 0.7	1.4 ± 1.0	0.41
Arterial wall thickening	0.7 ± 0.8	0.8 ± 0.5	0.64
Arteriolar hyaline change	1.1 ± 1.1	0.6 ± 1.2	0.41

Abbreviations: PSL = prednisolone, LST = losartan.

### Comparison of Efficiency of Therapy in Two Groups

**Figure 1:**
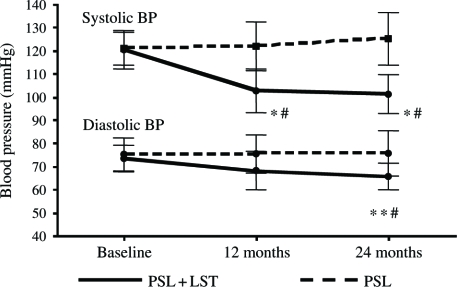
Blood pressure levels between the two groups. *Compared with baseline, *p* < 0.01; **compared with baseline, *p* < 0.05; ^#^comparison between the PSL + LST group and PSL-alone group, *p* < 0.05. Abbreviations: PSL = prednisolone; LST = losartan.

**Figure 2:**
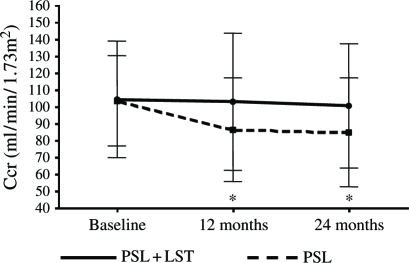
Creatinine clearance levels between the two groups. *Compared with baseline, *p* < 0.05. Abbreviation: Ccr=creatinine clearance.

**Figure 3:**
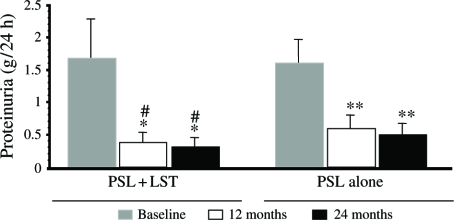
Proteinuria levels between the two groups. *Compared with baseline, *p* < 0.01; **compared with baseline, *p* < 0.01; ^#^comparison between the PSL + LST group and PSL-alone group, *p* < 0.05. Abbreviations: PSL = prednisolone; LST = losartan.

Primary end points could be analyzed in 38 of the 40 patients at the end of the 24 months period. Two patients in the PSL + LST group developed postural hypotension. After 24 months of treatment, 24-h urinary protein excretion decreased by 50+% in 18 of the 20 patients (90%) in the PSL + LST group and in 15 of 18 (83%) in the PSL group compared with baseline values (*p* < 0.05). The serum creatinine of four patients in the PSL group increased ≥50%. Table 3 shows the clinical follow-up data for the two groups. The SBP was significantly decreased at 12 and 24 months of treatment in the PSL + LST and PSL groups compared with baseline (both *p* < 0.01), and DBP was significantly decreased at 24 months of treatment in the PSL + LST group compared with baseline and the PSL group (*p* < 0.01). However, DBP did not change significantly in the PSL group (see Figure 1). The level of serum creatinine remained unchanged in the PSL + LST group, but was significantly increased in the PSL group at 12 and 24 months of treatment compared with baseline (*p* < 0.05, respectively). The Ccr fell significantly at 12 and 24 months of treatment in the PSL group compared with baseline (*p* < 0.05, respectively) but remained unchanged in the PSL+LST group. The Ccr was lower in the PSL than in the PSL+LST group at 12 and 24 months of treatment (see Figure 2). Proteinuria significantly decreased at 12 and 24 months of treatment in both groups compared with baseline (*p* < 0.05 in both groups, respectively), and PSL+LST was more effective than PSL alone at 12 and 24 months (*p* < 0.05, respectively; Figure 3). Total protein levels significantly increased compared with the baseline at 12 and 24 months of treatment in the PSL+LST group, but remained unchanged in the PSL group (see Table 3).

**Table 3:** Comparison of clinical follow-up data between patient groups

Characteristic	Group	Baseline	12 months	24 months
BMI, kg/m^2^	PSL + LST	21.2 ± 3.1	21.4 ± 3.2	21.4 ± 3.1
	PSL	19.9 ± 1.7	20.1 ± 2.0	20.1 ± 1.8
Systolic BP, mmHg	PSL + LST	120 ± 8	102 ± 9*#	101 ± 8*#
	PSL	121 ± 7	121 ± 11	125 ± 12
Diastolic BP, mmHg	PSL + LST	73 ± 6	68 ± 8	65 ± 6*#
	PSL	75 ± 7	75 ± 8	75 ± 10
K, mEq/L	PSL + LST	4.3 ± 0.3	4.4 ± 0.2	4.4 ± 0.3
	PSL	4.4 ± 0.2	4.4 ± 0.3	4.4 ± 0.4
sCr, mg/dL	PSL + LST	0.8 ± 0.2	0.8 ± 0.2	0.8 ± 0.2
	PSL	0.7 ± 0.1	0.9 ± 0.1**	0.9 ± 0.2**
Ccr ml/min/1.73 m^2^	PSL + LST	104 ± 36	102 ± 42	100 ± 38
	PSL	103 ± 28	86 ± 32**	84 ± 34**
Proteinuria, g/24 h	PSL + LST	1.6 ± 0.6	0.3 ± 0.1*#	0.3 ± 0.1*#
	PSL	1.6 ± 0.4	0.5 ± 0.2**#	0.5 ± 0.1**#
Total protein, g/dL	PSL + LST	6.0 ± 0.4	6.6 ± 0.4*	6.7 ± 0.4*
	PSL	6.2 ± 0.2	6.3 ± 0.2	6.4 ± 0.3

Abbreviations: BMI = body mass index, BP = blood pressure, BUN = blood urea nitrogen, sCr = serum creatinine, Ccr = creatinine clearance.

* Compared with baseline, *p* < 0.01.

** Compared with baseline, *p* < 0.05.

# Comparison between PSL + LST and PSL-alone, *p* < 0.05.

## DISCUSSION

The present study compared the efficiency of combined therapy with PSL (30 mg/day induction; 5 mg/day maintenance) + LST (50 mg/day) and PSL therapy alone in patients with IgAN and moderately severe histological characteristics defined as glomerular Katafuchi's scores of 4 to 7.[14] Our preliminary results demonstrated that LST decreases proteinuria in IgAN receiving PSL. Notably, the more rigorous BP control achieved with LST (mean BP of 101/65 mmHg) might have inhibited the reduction in GFR in patients with IgAN compared with those under normal BP control, whereas PSL alone did not. This finding suggests that the combination of PSL + LST can be safely administered to patients with IgAN. Our study indicates that PSL + LST more effectively reduces proteinuria and protects renal function in patients with IgAN than PSL monotherapy. Because Katafuchi et al.[14] revealed evidence of a mild protective effect of dipyridamole on renal survival in patients with IgAN, we administered dipyridamole to all patients in the present study.

Some trials were considered to be of sufficient quality to be included in a recent meta-analysis of immunosuppressive treatments for IgAN.[16] That analysis revealed that corticosteroid therapy might reduce proteinuria and the risk of end-stage renal disease (ESRD). Pozzi et al.[17] recently found from a ten-year follow-up of a large Italian study that corticosteroids confer impressive benefits in terms of reducing proteinuria and preventing ESRD. However, their high-dose corticosteroid regimen, (three courses of intravenous methylprednisolone, 1 g/day, for three consecutive days plus oral prednisolone, 0.5 mg/kg, on alternate days for six months), is regarded by many physicians as likely to carry considerable toxicity, even though none has been reported by investigators.[18] In our study, although the serum creatinine of four patients in the PSL group increased ≥50%, no severe side effects were observed in the PSL and PSL + LST group. Notably, a renin-angiotensin system blockade has only been applied to a minority of patients, and although equally distributed among the participants, the achieved BP was not in line with current recommendations.[18] Moreover, a recent randomized, prospective, controlled study of corticosteroids (20 mg/day induction; 5 mg/day maintenance) of Japanese patients found only a modest reduction in proteinuria with no difference in renal survival.[11] Katafuchi et al.[11] had been described that the absence of renoprotection might be to an insufficient dose of PSL, without mentioning of the extent of BP control.

Some specific evidence from patients with IgAN justifies strict BP control: in one small randomized, prospective, controlled study, a mean BP of 129/70 mmHg stabilized Ccr over three years, whereas the mean Ccr declined in patients with a BP of 136/76 mmHg at a rate of 13 mL/min over three years.[19] Therefore, Barratt et al.[18] proposed that corticosteroids should be considered only when proteinuria continues (>1 g/24 h) despite tight BP control (<125/75 mmHg) and with a maximal reninangiotensin system blockade. Although we stabilized BP at a mean of 101/65 mmHg Ccr over two years with PSL + LST, a definite conclusion from this preliminary study of few patients within in a short observation period cannot be drawn.

In conclusion, this preliminary study indicated that therapy with PSL and LST is more effective than that with PSL alone in reducing proteinuria and protecting renal function in patients with moderately proteinuric IgAN and moderate histological features. Future studies will deepen understanding about this important clinical correlation, and specifically allow determination of the ideal target range of BP and dose of PSL.
